# Surgery

**Published:** 1911-06

**Authors:** H. G. Kyle


### SURGERY.

**Treatment of Stone in the Bladder.**—In his *Lettsomian Lectures*[2] Mr. W. F. Haslam traces the history of operations for stone from the earliest times, giving accounts of the various procedures adopted in the past. In the last lecture he sets forth the methods at present in vogue amongst the surgeons of this country. In order to ascertain their present practice, he sent a series of questions to the surgeons attached to the principal English hospitals. The answers to these questions are interesting as representing the opinion and practice of the surgeons of this country at the present date. He received about 240 replies. The first fact that strikes us on examining these replies is that suprapubic lithotomy is now the operation of choice for all cases young or old, simple or complicated. About 67 per cent. state this to be their universal practice. A few years ago lithotrity was the favourite method ; now only 12 per cent. express themselves in its favour as a routine operation. The opinion generally held is, that unless you see enough of stone to become proficient in lithotrity it is better to cut. Another very striking fact is that the prevalence of stone in the bladder is markedly decreasing—it is now a rare disease in this country. This statement is made by surgeons in all parts of England.

From Norwich, at one time a great centre for stone, a surgeon writes : " We scarcely get any cases of stone in the bladder

[1] *Loc. cit.* [2] *Brit. M. J.*, 1911, i. 347, 435, 548.

now, though fairly common in the kidney. This is a change, as I can remember, about thirty years ago, seeing three lithotomies in a morning." The general surgeon, then, has now but little chance of becoming an expert lithotritist.

When the method of performing lithotrity was so greatly improved by Bigelow in 1878, a suprapubic opening of the bladder was still a serious undertaking, and crushing came into considerable favour for the next twenty-five years or so. The comparatively slight risk now attached to cutting operations, the greater precision and rapidity of cystotomy, and the few opportunities of becoming an adept at crushing, leave little doubt but that suprapubic cystotomy must be at the present time the operation of choice. It is, moreover, suitable for all cases of stone. The great majority of those who practise lithotrity allow that crushing is not suitable for certain conditions, such as enlarged prostate, cystitis, extreme youth and great size or hardness of the stone. A considerable number of stones are associated with enlargement of the prostate and cystitis, and therefore not suitable for crushing, except in the opinion of very few. Of 224 who replied to the question as to the method adopted in prostatic enlargement, only sixteen mentioned crushing, and they were only prepared to attempt it "if possible."

A more detailed analysis of the replies to Mr. Haslam's question is worthy of study.

QUESTION 1.—What operation do you usually perform for the removal of a stone before the age of puberty?

221 replies, which he groups as follows: suprapubic lithotomy, 144; lithotrity for small stones, otherwise suprapubic, 35; lithotrity if possible, 9; lithotrity, 7; suprapubic as a rule, sometimes lithotrity, 9; after four years lithotrity, before, suprapubic, 2; after seven years lithotrity, before suprapubic, 1; lithotrity almost invariably, but in large hard stones in very small children suprapubic, 6; lithotrity if no permanent cystitis, 2; lithotrity or suprapubic, 1; lithotrity or lateral lithotomy, if the stone is large, suprapubic, 1; median lithotomy, 1; lateral lithotomy, but lately suprapubic, 1; suprapubic, and if the stone re-forms median or lateral lithotomy, 1; lateral lithotomy, rarely lithotrity, 1.

It is clear from these figures that the suprapubic operation is the one usually performed. Only 3 per cent. refer to lithotrity as the method adopted without reference to any contra-indications. The perinæal method is mentioned, but is clearly almost obsolete.

QUESTION 2.—What operation do you usually perform for the removal of a stone from the male bladder after puberty, when there is no evidence of prostatic enlargement?

240 replies as follows: suprapubic lithotomy, 116; lithotrity 29; lithotrity as a rule, 28; lithotrity for small stones, otherwise suprapubic, 51; almost always suprapubic, sometimes lithotrity, 8; lateral lithotomy occasionally, especially when the urine is foul, 2; with foul cystitis suprapubic lithotomy in two stages, 1; formerly lateral lithotomy, of late suprapubic, 1; if the stone is large median urethrotomy with crushing, 1; if the stone is small median or lateral lithotomy, if large suprapubic, 1; lithotrity except with sacculation or sepsis, then suprapubic, 1; lithotrity if possible, if not suprapubic lithotomy, 1.

These returns show that surgeons are prepared to consider the advisability of performing lithotrity in the adult more frequently than in boys, but the number of those who practise the suprapubic operation is in excess of those who use lithotrity, and of these a large number reserve it for small stones, doing the suprapubic operation for large ones.

Question 3.—What method do you adopt when there is evidence of prostatic enlargement?

224 replies: suprapubic lithotomy, 202; lithotrity if possible, 16; other modifications, 6

For these cases, then, suprapubic lithotomy is the method adopted by almost all surgeons. Of the sixteen who advocate lithotrity all make reservations to their statement. Curiously only two make any reference to removing the prostate at the same time as the stone. Possibly such reference may have been looked upon as irrelevant, information on this point not being asked for in the question.

If we consult the principal surgical textbooks we find a somewhat different view: they all more or less recommend lithotrity. A. T. Cabot, who writes the article on stone in Keen's *Surgery*, says that litholapaxy should be employed in all cases when the urethra will admit instruments. If the stone is too large to be grasped by the lithotrite, or too hard to be crushed, the attempt should be abandoned, and a cutting operation resorted to. "Litholapaxy," he says, "is the operation of choice in adults."

H. G. Kyle.

